# Relationship of Beta-Human Chorionic Gonadotropin to Ectopic Pregnancy Detection and Size

**DOI:** 10.5811/westjem.18396

**Published:** 2024-05-03

**Authors:** Duane M. Eisaman, Nicole E. Brown, Sarah Geyer

**Affiliations:** * University of Pittsburgh; † Chatham University; ‡ University of Pittsburgh Medical Center

## Abstract

**Introduction:**

Ectopic pregnancies are a significant cause of morbidity and mortality in the first trimester of pregnancy. Hospital protocols requiring a specific beta-human chorionic gonadotropin (β-hCG) level to qualify for diagnostic testing (pelvic ultrasound) can delay diagnosis and treatment. In this study we sought to determine the relationship between β-hCG level and the size of ectopic pregnancy with associated outcomes.

**Methods:**

We performed a retrospective case review of patients diagnosed with ectopic pregnancy in an urban, academic emergency department specializing in obstetrical care, from January 1, 2015–December 31, 2017. Variables extracted included presentation, treatment, adverse outcomes, and rates of rupture.

**Results:**

We identified 519 unique ectopic pregnancies. Of those ectopic pregnancies, 22.9% presented with evidence of rupture on ultrasound, and 14.4% showed evidence of hemodynamic instability (pulse >100 beats per minute; systolic blood pressure <90 millimeters of mercury; or evidence of significant blood loss) on presentation. Medical management outcomes were as follows: of 177 patients who received single-dose methotrexate, 14.7% failed medical management and required surgical intervention; of 46 who received multi-dose methotrexate, 36.9% failed medical management and required surgical intervention. Ultimately, 55.7% of patients required operative management of their ectopic pregnancy. Mean β-hCG level at initial presentation was 7,096 milli-international units per milliliter (mIU/mL) (SD 88,872 mIU/mL) with a median of 1,289 mIU/mL; 50.4% of ectopic pregnancies presented with β-hCG levels less than the standard discriminatory zone of 1,500 mIU/mL. Additionally, 44% of the patients who presented with evidence of rupture had β-hCG levels less than 1,500 mIU/mL. Comparison of size of ectopic pregnancy (based on maximum dimension in millimeters) to β-hCG levels revealed a very weak correlation (r = 0.144, ***P*** **< .001**), and detection of ectopic pregnancies by ultrasound was independent of β-hCG levels.

**Conclusion:**

Levels of β-hCG do not correlate with the presence or size of an ectopic pregnancy, indicating need for diagnostic imaging regardless of β-hCG level in patients with clinical suspicion for ectopic pregnancy. Almost one-sixth of patients presented with evidence of hemodynamic instability, and approximately one quarter of patients presented with evidence of rupture requiring emergent operative management. Ultimately, more than half of patients required an operative procedure to definitively manage their ectopic pregnancy.

Population Health Research Capsule What do we already know about this issue?

*Beta-human chorionic gonadotropin (β-hCG) levels and the discriminatory zone cutoffs are used to determine ultrasound-ordering algorithms at many hospitals.*

What was the research question?

*Do β-hCG levels correlate with size of an ectopic pregnancy and/or rate of treatment failure?*

What was the major finding of the study?

*50.4% of ectopic pregnancies, and 44% who had rupture, had β-hCG levels less than 1,500 mIU/mL. The ectopic pregnancy β-hCG levels only very weakly correlated (r = 0.144, P < .001) with ectopic size.*

How does this improve population health?

*β-hCG levels should not be a factor in ordering transvaginal ultrasound in a patient with suspected ectopic pregnancy.*

## INTRODUCTION

Ectopic pregnancies are not a rare occurrence, affecting approximately 2% of all pregnancies.^1^^–^^3^ There is no centralized system to monitor rates of ectopic pregnancy; thus, the true incidence is likely higher than this estimate.^2^ Ectopic pregnancy is a leading cause of morbidity and mortality in the first trimester of pregnancy. Medical management requires more from a patient in terms of follow-up (multiple blood draws, ultrasounds, and appointments) when compared to surgical management; between 12–24% of patients will fail medical management and ultimately require surgical management.^4^

The classic presentation of an ectopic pregnancy is unilateral pelvic pain with vaginal bleeding in the presence of a positive pregnancy test. Risk factors (present in 50% of those with ectopic pregnancy) include prior ectopic pregnancy; history of pelvic inflammatory disease or pelvic surgery; assisted reproductive technology for conception; age >35 years; tobacco use; intrauterine diethylstilbestrol exposure; and presence of an intrauterine device at the time of conception.^1^^,^^2^ About 96% of ectopic pregnancies will occur within the adnexa; rarer locations include the cervix, cesarean section scars, ovaries, and abdominal cavity.^1^^,^^3^^,^^5^

Diagnostic workup for suspected ectopic pregnancy includes bloodwork to check for beta-human chorionic gonadotropin (β-hCG) levels, Rh type, hemoglobin level, and transvaginal ultrasound to visualize location of the pregnancy (intra- vs extrauterine). The published discriminatory zone for β-hCG levels (1,500–4,000 milli-international units per milliliter [mIU/mL]) can be used to aid for correlation to expected ultrasound findings. Patients with a β-hCG >3,500 mIU/mL should have findings on ultrasound that demonstrate the location of the pregnancy (intrauterine [gestational sac plus yolk sac within the endometrial cavity] vs ectopic [lack of intrauterine pregnancy with an extrauterine mass with sonographic characteristics consistent with ectopic pregnancy]).^1^ While the discriminatory zone is helpful to determine when an intrauterine gestation should be seen on ultrasound, signs of an ectopic pregnancy may be visible at significantly lower β-hCG levels.^6^ In this study we sought to determine whether β-hCG levels correlate with the size of an ectopic pregnancy as well as the rate of treatment failure of ectopic pregnancy.

## METHODS

We performed a retrospective case review of patients seen in an urban, academic ED housed in a tertiary- care facility specializing in obstetrical care. Cases occurred between January 1, 2015–December 31, 2017, for patients who were diagnosed with an ectopic pregnancy. This study was approved by the institutional review board; participant consent was not required. The criterion for inclusion was a diagnosed ectopic pregnancy in the chart and/or on ultrasound, identified by searching billing codes for “Ectopic Pregnancy,” “Ectopic Pregnancy, Other,” “Abdominal Pregnancy,” “Tubal Pregnancy,” “Ectopic Pregnancy, Nonspecific,” and “Ovarian Pregnancy.” This search yielded 1,265 visits during the research period with 519 unique cases of ectopic pregnancy (where each case could have multiple associated visits). Patients of all ages were included in the study if they met the inclusion criterion. Exclusion criteria was loss of patient to follow-up after initial presentation (thus making it impossible to confirm final diagnosis of ectopic pregnancy and/or outcomes related to treatment) or if the patient was ultimately found to have a diagnosis other than ectopic pregnancy (ie, intrauterine pregnancy). For the 519 cases of ectopic pregnancy, data extracted from each chart included the following variables: presentation (including initial β-hCG levels and ultrasound findings [size of ectopic pregnancy, evidence of rupture, and/or fetal heartbeat]); treatment (expectant, medical, and/or surgical); and treatment outcomes (successful, failure, or rupture). Data was extracted by two individuals; the principal investigator (**xx**) reviewed the extracted data every five cases to ensure consistency of data extraction. We compared data using standard means, the Pearson correlation coefficient, and Student *t*-testing using Excel (Microsoft Corp, Redmond, WA). For charts where data was incomplete (for example, missing ultrasound evidence of ectopic pregnancy), we included the chart in the analyses where data could be extracted and we excluded data from the analyses where it was missing.

## RESULTS

The dataset contained 519 unique ectopic pregnancies presenting to the ED during a three-year period (Table). The average age of the subjects at presentation was 29.39 years with near equal racial distribution between self-reported Black and White. Evidence of ectopic rupture was present in 22.9% of cases on presentation, and 14.4% had evidence of hemodynamic instability. A heartbeat was detected 9.1% of the time, which per current American College of Obstetricians and Gynecologists (ACOG) recommendation is a relative contraindication for treatment with methotrexate.^1^ Subjects receiving single-dose methotrexate treatment had a failure rate of 14.7%. Subjects who received multi-dose methotrexate (generally because they did not meet ACOG requirements for single-dose methotrexate treatment) had a failure rate of 36.9%. The β-hCG levels for both ruptured and non-ruptured ectopic pregnancies were similar.

**Table.** Age of patient in years, reported race, rate of ectopic rupture at presentation, unstable at presentation (heart rate >100 bpm, systolic blood pressure <90 mmHg, or documentation of instability), rate of methotrexate failure after treatment, rate of live ectopic at presentation, β-hCG levels for visualized ectopic, β-hCG levels for ruptured vs non-ruptured ectopic, and greatest diameter of visualized ectopic.

Variable assessed	Findings
Age (Years)	29.39 (SD 4.42)
Race (May include more than one)	
White	51.8%
Black	40.8%
Asian	3.6%
American Indian/Native American	0.6%
No response	2.7%
Ruptured at presentation	22.9%
Unstable at presentation	14.4%
Methotrexate failure rate	
Single dose	14.7%
Multi dose	36.9%
Live ectopic at presentation	9.1%
β-hCG levels (mIU/mL)
Ruptured	7,005 (SD 8,949)
Non-ruptured	7,609 (SD 8,773)
*T*-test	*P* = 0.51
β-hCG for visualized ectopic pregnancy (mIU/mL; n = 456)	
Mean	4,964 (SD 12,217)
Median	1,209
Greatest diameter of visualized ectopic pregnancy (mm; n = 456)	
Mean	24 (SD 14.3)
Median	21

*β-hCG*, beta-human chorionic gonadotropin; *mlU/ML*, milli-international units per milliliter; *mm*, millimeter.

Greatest dimension of ectopic pregnancy (diameter in millimeters [mm]) was correlated with β-hCG level (milli-international units per milliliter [mIU/mL]) for each patient (Figure). We excluded from this analysis patients who did not have an extrauterine mass visualized on ultrasound as dimensions of ectopic size were unknown. Mean β-hCG for this group of 456 subjects was 4,964 mIU/mL (SD 12,217; 95% confidence interval [CI] 3,793–6.135), and median β-hCG was 1,209 mIU/mL; mean greatest diameter was 24 mm (SD 14.3; 95% CI 22.69–25.31) and median was 21 mm. The Pearson correlation coefficient (r-value) of this group was r = 0.144 (*P* < .001), indicating a very weak correlation between β-hCG level and greatest dimension of ectopic pregnancy. We also calculated the Pearson correlation for β-hCG compared to ectopic volume (in mm^3^); the results were not statistically significant.

**Figure.**
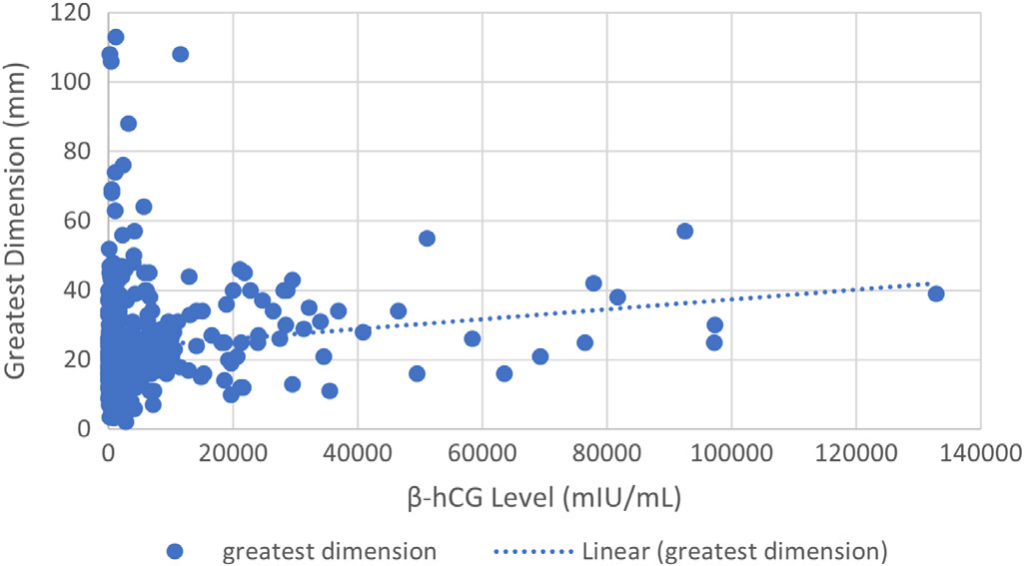
β-hCG level in relation to the greatest dimension of ectopic pregnancy (n = 456). Pearson correlation between β-hCG level and greatest dimension was r = 0.144 (*P* < .001), demonstrating a very weak correlation. *β-hCG*, beta-human chorionic gonadotropin; *mm, millimeters; mIU/mL,* milli-international units per milliliter.

## DISCUSSION

Nearly 25% of patients with an ectopic pregnancy in this study presented with evidence of rupture, and about 15% of patients presented with evidence of hemodynamic instability, both scenarios requiring emergent surgical treatment. The ectopic rupture rate seen in this study was greater than prior reported rates of 15%, although, interestingly, less than 10% of ruptured ectopic pregnancies in the study required a blood transfusion (data not shown), which is similar to previous published data of 8.7%.^7^^,^^8^

The majority of visualized ectopic pregnancies in the current study had β-hCG levels below the traditional 1,500 mIU/mL discriminatory zone, with the lowest β-hCG level in the study being 9 mIU/mL (which had sonographic evidence of an ectopic pregnancy). The β-hCG levels were similar between ruptured and non-ruptured ectopic pregnancies. Prior studies have also demonstrated low β-hCG levels in ectopic pregnancies, with levels as low as <10 mIU/mL documented.^9^ While ectopic pregnancies typically have lower β-hCG levels compared to intrauterine pregnancies, the presence of very low β-hCG levels in documented ectopic pregnancy cases is striking.^9^ One study found that 41% of ectopic pregnancies had a β-hCG level <2,000 mIU/mL at the time of diagnosis, and approximately 9% (18 of 204 cases) had a β-hCG level under 100 mIU/mL.^9^ These findings combined with our results (50.4% of cases had a β-hCG <1,500 mIU/mL, and 8.5% had a level <100 mIU/mL) emphasize the need to consider and work up suspected ectopic pregnancies fully at the time of presentation, regardless of serum β-hCG level, to avoid missing the diagnosis of ectopic pregnancy. Thus, discriminatory cutoffs of β-hCG levels should not be a determining factor when ordering transvaginal ultrasonography to evaluate for ectopic pregnancy.^10^

We found a very weak correlation between β-hCG and size of ectopic pregnancy, when looking at greatest dimension, and no correlation of β-hCG to volume of ectopic pregnancy. These findings are in stark contrast to prior studies, where β-hCG levels and ectopic pregnancy volume were found to be strongly correlated.^9^ Therefore, this study indicates that β-hCG levels may have less predictive value for estimating the size or volume of an ectopic pregnancy than previously thought, further strengthening the need for transvaginal ultrasound attainment regardless of β-hCG level.

Post-methotrexate failure or rupture is not uncommon; about 20% of patients in the current study ultimately required surgical management (higher than the 10.2% reported in previous studies).^11^ This increased rate of post-methotrexate failure might be related to study location (tertiary-care center for obstetrical care), patient delay in seeking care, or higher rate of cases necessitating multiple-dose methotrexate treatment (due to treatment at a tertiary-care center), although any conclusion regarding these variables is difficult. Patients receiving methotrexate for ectopic pregnancy with complaints of new or worsening abdominal pain or increased vaginal bleeding should be evaluated for potential ectopic pregnancy rupture.

## LIMITATIONS

The study took place at a single-site academic center, where the population of the ED could have been skewed due to location (urban), referral center status (tertiary care for obstetrical care), and higher acuity level of care compared to many hospitals. In addition, patient encounters took place between 2015–2017 using ACOG guidelines published in 2008; however, current ACOG guidelines (released in 2018) are similar to those used during the timeframe of patient care; thus, any potential effect on the data was negligible.^1^^,^^12^

## CONCLUSION

In the setting of a positive pregnancy test, pelvic pain and/or vaginal bleeding should prompt a complete workup for ectopic pregnancy to include Rh status, hemoglobin, β-hCG level, and transvaginal pelvic ultrasound (regardless of β-hCG level). Clinicians must consider the ongoing risk of ectopic pregnancy rupture after methotrexate treatment. Finally, patients may be lost to follow-up and have an untreated ectopic pregnancy, which can lead to significant morbidity and/or mortality.
